# Lived Experiences and Perceptions of Healthy Lifestyles Among Kichwa Kayambi Indigenous Adults: An Interpretative Qualitative Study in the Community of Pijal

**DOI:** 10.3390/healthcare14193250

**Published:** 2026-10-01

**Authors:** Karen Jaramillo-Jácome, Katty-Elizabeth Cabascango, Melisa Chacón-Guerra

**Affiliations:** Faculty of Health Sciences, Universidad Técnica del Norte, Ibarra 100150, Ecuador; kecabascango@utn.edu.ec (K.-E.C.); mgchacong@utn.edu.ec (M.C.-G.)

**Keywords:** healthy lifestyles, Indigenous population, Andean worldview, transcultural nursing, qualitative research

## Abstract

Background: Healthy lifestyles in Indigenous populations cannot be understood solely based on biomedical categories; they require approaches that consider cultural meanings, material conditions, and community relationships. Objective: This study aimed to understand the lived experiences and perceptions of healthy lifestyles among Kichwa Kayambi Indigenous adults in the community of Pijal, Imbabura, Ecuador. Methods: An interpretative qualitative study was conducted based on 20 in-depth semi-structured interviews with Indigenous adults (15 women, 5 men; ages 25–72 years). The analysis followed a hybrid thematic approach with double coding and analyst triangulation. Leininger’s Theory of Diversity and Universality of Cultural Care and Pender’s Health Promotion Model were used as sensitizing concepts. Results: Three interrelated categories emerged. First, responsibility for health care is assumed as a daily decision, conditioned by economic barriers and facilitated by community cohesion. Second, life habits (physical activity linked to agricultural work, diet based on local products, rest according to work rhythms, ceremonial uses of tobacco and alcohol) articulate biomedical and traditional knowledge. Third, the Andean worldview integrates health with spirituality, the relationship with Pachamama, the use of medicinal plants, and intergenerational memory. Conclusions: A healthy life in this community is configured within a relational fabric where personal responsibility, daily habits, and the Andean worldview overlap in practices such as cultivating food, walking during work, preparing herbal remedies, and giving thanks for the harvest. A situated understanding of these meanings is essential for developing culturally congruent nursing interventions.

## 1. Introduction

### 1.1. Healthy Lifestyles in Intercultural Contexts

Healthy lifestyles encompass everyday practices related to diet, physical activity, rest, emotional well-being, social relationships, and health care. Although frequently framed as individual choices, these practices are shaped by family, economic, social, cultural, and environmental conditions [1,2]. In Indigenous contexts, health and well-being may also be inseparable from relationships with family, community, territory, nature, culture, and spirituality [3]. Culture therefore provides a framework of knowledge, values, practices, and symbols through which the body, health, illness, and care are interpreted. Accordingly, this study uses healthy lifestyles as an orienting rather than as a prescriptive concept to explore how participants experience and interpret their care practices within their sociocultural context [4,5].

Within the Andean worldview, well-being reflects a dynamic balance among the individual, family, community, nature, and spirituality. Principles such as reciprocity (ayni), complementarity, and correspondence, together with collective practices such as the minga, locate the individual within a network of interdependence. Illness may therefore be understood as a disruption of the bodily, social, natural, or spiritual balance that sustains life [6,7].

### 1.2. Latin American and Ecuadorian Context

Across Latin America, previous research indicates that Indigenous peoples are experiencing cultural, economic, dietary, and territorial transformations associated with changing diets, reduced physical activity embedded in daily work, and greater exposure to industrialized foods [8,9]. Food security also has cultural and emotional dimensions: experiences of scarcity, access to food, and family and community meanings shape perceptions of risk and care responses [10,11].

The relationship between territory and health becomes especially important in the context of environmental degradation and climate change. Deforestation, territorial loss, and ecosystem disruption affect livelihoods, cultural practices, and physical and mental health; they may also weaken symbolic and spiritual bonds associated with Sumak Kawsay, or Good Living, understood here as a relational way of life grounded in balance and reciprocity among individuals, community, territory, nature, and spirituality [12].

In Ecuador, studies conducted in rural and Indigenous populations have documented problems of food insecurity, economic inequality, nutritional alterations, and difficulties in preserving certain care practices in contexts of insecurity and dietary transition [13,14,15]. However, other studies developed among priority care groups and populations living in poverty have described problems related to diet, physical well-being, mental health, and quality of life.

In this context, local agricultural production, consumption of traditional foods, use of medicinal plants, and knowledge transmitted by mothers, fathers, grandparents, and other community members continue to form part of care practices [14,16]. Nevertheless, this knowledge does not remain unchanged; it is transformed through experiences, generational changes, economic conditions, and contact with health services. Therefore, its understanding requires listening to the voices of those who practice it and observing the circumstances in which it acquires meaning.

As the core of society, the family constitutes one of the main spaces for learning to eat, work, relate to nature, and care for health. Kinship networks, social support, and community forms of organization can strengthen the capacity to face shared needs and sustain certain care practices [17,18]. From this perspective, well-being depends not only on each individual’s will but also on cooperation, reciprocity, access to resources, and the possibility of participating in decisions that affect community life (Sumak Kawsay).

### 1.3. Knowledge Gap and Justification for the Study

Although the communities themselves possess extensive knowledge regarding their forms of care, the scientific literature has documented in a limited manner how Kichwa Kayambi adults from Pijal experience and interpret a healthy life within their sociocultural context. In particular, there remains a need to understand how everyday practices, intergenerational knowledge, family relationships, community organization, and the Andean worldview are articulated [4,5].

Leininger’s Theory of Culture Care Diversity and Universality constituted the principal interpretive framework. This theory makes it possible to distinguish practices that should be preserved, practices that may be accommodated or negotiated, and situations that require repatterning in order to protect well-being through culturally congruent care [19,20]. Complementarily, Pender’s Health Promotion Model contributed concepts regarding prior experiences, beliefs, and interpersonal and situational influences [21,22]. Both frameworks were employed as sensitizing concepts, rather than as rigid categories imposed upon the narratives.

Pijal, a Kichwa Kayambi community in the province of Imbabura, offers a pertinent context due to its agricultural activities and forms of community organization [23]. The study did not assume that local practices were homogeneous or immutable; rather, it attended to their continuities, transformations, and generational, familial, and social differences.

The objective was to understand the lived experiences and perceptions regarding healthy lifestyles among Kichwa Kayambi Indigenous adults from Pijal, Imbabura, Ecuador. The meanings attributed to personal, familial, and community responsibility, everyday well-being practices, and the traditional knowledge and meanings that the Andean worldview confers upon the body, health, illness, and a healthy life were explored.

## 2. Materials and Methods

### 2.1. Study Design

An interpretative qualitative study was conducted based on in-depth semi-structured interviews and analyzed using hybrid thematic analysis. The analysis was guided by principles of interpretive ethnography, which emphasize the understanding of cultural meanings from the actors’ perspectives, situating narratives within their community context. However, the design does not correspond to a classical ethnography, as data production was limited to interviews, without participant observation or prolonged immersion in the field. This orientation is consistent with interpretive ethnography, whose exponents recognize that all cultural description is a situated interpretation, constructed through the interaction between the researcher and the participants [24,25,26]. The completed COREQ checklist is provided in Supplementary Material S1.

This approach was appropriate for understanding the lived experiences and perceptions that Indigenous adults in the community of Pijal attribute to healthy lifestyles, by allowing their narratives on traditional knowledge, family and community relationships, and the bond with the territory to be situated within the cultural framework from which they were narrated [27,28]. From this perspective, a healthy life was not understood as a set of isolated behaviors, but as a dimension of cultural identity, linked to family, community, nature, and the spiritual world characteristic of the Andean worldview.

The study was developed under the principles of naturalistic inquiry, aimed at understanding meanings within the context in which experiences were constructed, without deliberate manipulation of community conditions [29].

Madeleine Leininger’s Theory of Diversity and Universality of Cultural Care constituted the main interpretative reference. Complementarily, Nola Pender’s Health Promotion Model was considered. Both frameworks were used as sensitizing concepts and not as rigid analytical categories established prior to the analysis.

### 2.2. Study Setting

The research was conducted in the Community of Pijal, belonging to the González Suárez parish, Otavalo canton, Imbabura province, Ecuador, located approximately 14 km south of the city of Otavalo and 85 km north of Quito. Its inhabitants belong predominantly to the Kichwa Kayambi people. The main economic activities of the community are agriculture and livestock farming. Local documents describe forms of community organization and ancestral ethical principles such as ama shua (do not steal), ama llulla (do not lie), and ama quilla (do not be lazy). Pijal also possesses natural and cultural resources, including ancestral raised fields (camellones), native forest, the Rinconada Waterfall, the Apangora spring, and the Itambí River. Activities related to community tourism, gastronomy, traditional music, and crafts are also developed [23].

A current official population estimate specific to Pijal was not available. For contextual reference, the 2023 parish development plan reported 5630 residents in González Suárez Parish, which comprises Pijal and seven other communities in addition to the parish center [23].

This rural context allowed the narratives to be situated within their cultural, social, and territorial framework. It was not assumed that cultural practices or the meanings attributed to a healthy life were static, homogeneous, or shared equally by all community members. On the contrary, generational, familial, occupational, and social differences expressed by the participants were considered [30].

### 2.3. Participants

A total of 20 Kichwa Kayambi Indigenous adults from Pijal participated: 15 women and 5 men, across three age groups: 25–39, 40–59, and 60 years or older. Snowball or chain referral sampling was employed to facilitate access to eligible participants through existing community networks [31]. Prior to recruitment, a target of 20 participants was established, compatible with the available resources and the fieldwork period, with the aim of capturing variation by age, sex, occupation, family role, and community experience. This target was a sampling design decision and not a statistical threshold nor an anticipatory declaration of saturation.

Recruitment began with three deliberately diverse entry points—a community leader, an older adult woman, and a young farmer—in order to reduce dependence on a single social network. From these contacts, referrals were requested for other individuals who met the selection criteria, until the intended target was reached. Twenty eligible individuals were invited; all accepted, provided informed consent, and completed the interview. There were no refusals, withdrawals, or dropouts. The final number coincided with the target because recruitment was closed once the 20 interviews were completed, not because each chain produced a predetermined number of participants.

During concurrent analysis, the breadth and depth of the narratives and the recurrence of meanings were examined. The final conversations reiterated patterns already identified and did not introduce substantial variations; this observation was used to assess the adequacy of the corpus obtained, but not to retrospectively redefine the target of 20 interviews as a decision based on saturation or information power [32].

### 2.4. Data Production

Data production was carried out between March and June 2026, using in-depth semi-structured interviews, with a guide informed by the methodological considerations of ethnographic interviewing [33]. After collecting sociodemographic information, interviews began with the question: “Could you tell me a little about yourself and the role you play in your community?” Subsequently, the following was explored: “What does living healthily and feeling in balance in your daily life mean to you?” Probing questions were asked to request examples, clarify expressions, and expand on the meanings narrated.

The guide was developed using the E-VEVSA as an initial conceptual reference, without administering it as a scale or using its scores to classify participants [34]. Although the E-VEVSA was validated in a Spanish adult population and not in a Kichwa Kayambi population, its seven domains provided a preliminary map of everyday practices that was subjected to cultural adaptation and qualitative revision. The Andean worldview was incorporated as an eighth dimension, and the language, relevance, and sequence of topics were reviewed. The complete guide is attached as Supplementary Material S2 to make this adaptation transparent.

The 58 questions constituted a bank of potential stimuli and probes, not a list that had to be asked in full or in a fixed order. Each interview began with broad questions, and the interviewer selected follow-up questions according to the participant’s narrative; consequently, no uniform time was allocated to each dimension. Each individual participated in one interview lasting 60 to 90 min, a duration chosen to provide opportunities to delve into examples, clarify expressions, and explore the meanings that emerged during the conversation. Prior to fieldwork, five specialists in intercultural nursing, qualitative methodology, and public health reviewed the clarity, content, and cultural relevance of the guide.

Individual interviews were chosen instead of focus groups because the objective required reconstructing personal experiences and potentially sensitive practices—illness, economic resources, alcohol and tobacco consumption, and home remedies—under conditions of privacy. Moreover, this modality reduced the possible inhibition associated with community or family hierarchies. The study did not seek to analyze group interaction or to produce collective consensus, purposes for which a focus group would have been more appropriate.

All interviews were conducted by Katty-Elizabeth Cabascango, a woman and nursing professional who was a member of the research team, and who received training in qualitative methodology and semi-structured interviewing through in-person workshops. Karen Jaramillo-Jácome, a woman and the principal investigator, supervised the design, data production, and analysis, but did not conduct the interviews. Prior to the study, Katty-Elizabeth Cabascango had no familial, occupational, educational, or caregiving relationships with the participants.

Katty-Elizabeth Cabascango’s identity as a woman may have facilitated approach to the participants and trust during the interviews conducted in households; her health-related training and university status may also have positioned her as an external health agent. Initial contacts with participants and authorities made it possible to explain the study prior to the interviews, which were conducted solely by Katty-Elizabeth Cabascango and each participant in their homes, community spaces, or workplaces as agreed, in order to favor privacy and free expression.

All interviews were conducted and transcribed in full in Spanish, in accordance with the preference expressed by the participants and with what was agreed upon with the community authorities. Although several individuals were bilingual in Kichwa and Spanish, they communicated in Spanish during the interviews; therefore, it was not necessary to translate or interpret content from Kichwa into Spanish. Cultural terms without a direct conceptual equivalent, such as Pachamama, were preserved in their original form and explained in context. For publication in English, the selected quotations were translated directly from Spanish into English by Karen Jaramillo-Jácome. Their conceptual equivalence was independently verified against the original Spanish transcripts by Katty-Elizabeth Cabascango. Any differences were discussed and resolved by consensus. Back-translation into Kichwa was not applicable because all interviews had been conducted in Spanish. The interviews were audio-recorded with prior authorization, lasted between 60 and 90 min, and were not repeated. After each encounter, Katty-Elizabeth Cabascango recorded contextual, nonverbal, and reflective field notes.

The recordings were transcribed by the principal investigator and a nursing professional with a master’s degree in public health. Subsequently, both verified each transcription by re-listening to the original recording. The transcriptions were not returned to participants for review. To guarantee data fidelity, verification was carried out by re-listening to the original recordings. During anonymization, names, family relationships, specific locations, and other potentially identifying details were removed. Given the small-community setting, sociodemographic characteristics are reported only in aggregate and are not linked to quotation codes. Potentially identifying clinical details in quotations were omitted or generalized where necessary, without changing the analytical meaning of the narratives. Interviews were assigned consecutive alphanumeric codes (P01–P20).

### 2.5. Data Analysis

The analysis followed a hybrid thematic approach [24,25]. The dimensions of the interview guide and the Leininger and Pender frameworks were used as initial sensitizing concepts, while codes, subthemes, themes, and interpretative relationships were constructed from the participants’ narratives. This approach allowed the articulation of an initial conceptual orientation with the necessary openness to identify unanticipated meanings, avoiding the imposition of rigid categories onto the narrated experiences.

The analysis followed Braun and Clarke’s six-phase method of thematic analysis [24]: familiarization through repeated reading; initial coding of meaning units; development of provisional themes through comparison across interviews; review of themes against the full corpus; definition and naming of themes and subthemes; and interpretative integration of the findings using illustrative narrative excerpts.

**Figure A1:**
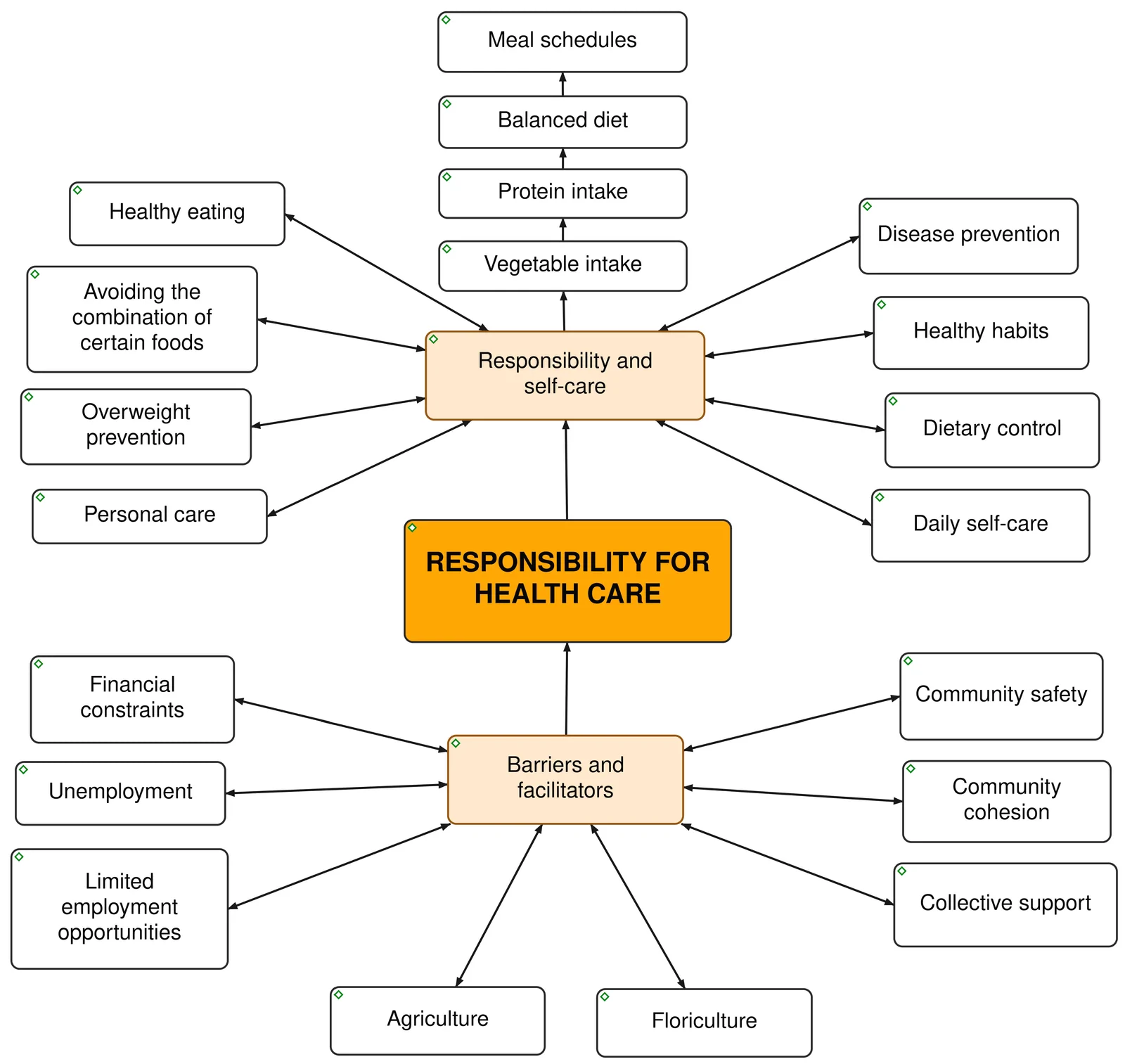
Category 1. Semantic network of responsibility in health care.

**Figure A2:**
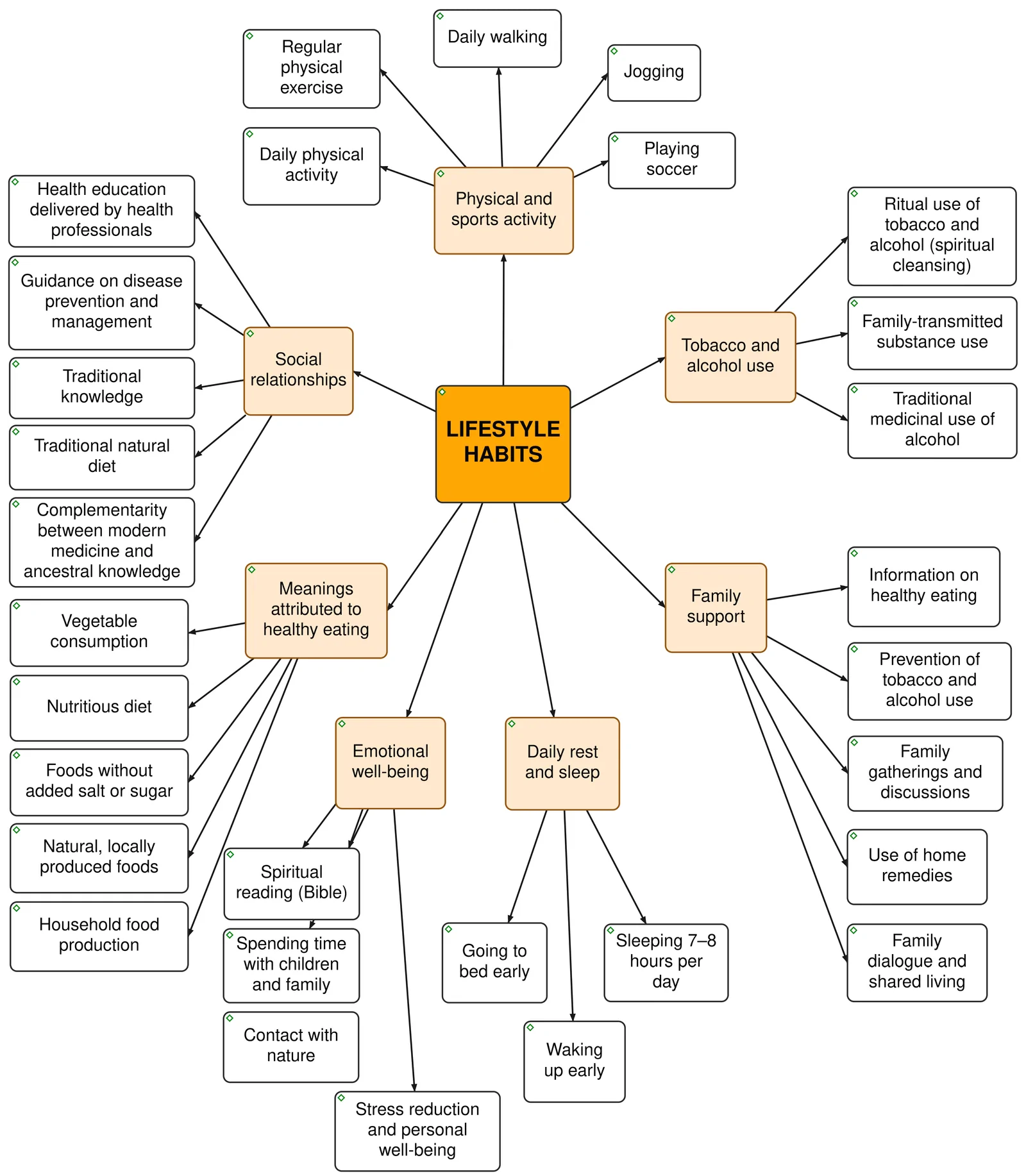
Category 2. Semantic network of life habits.

**Figure A3:**
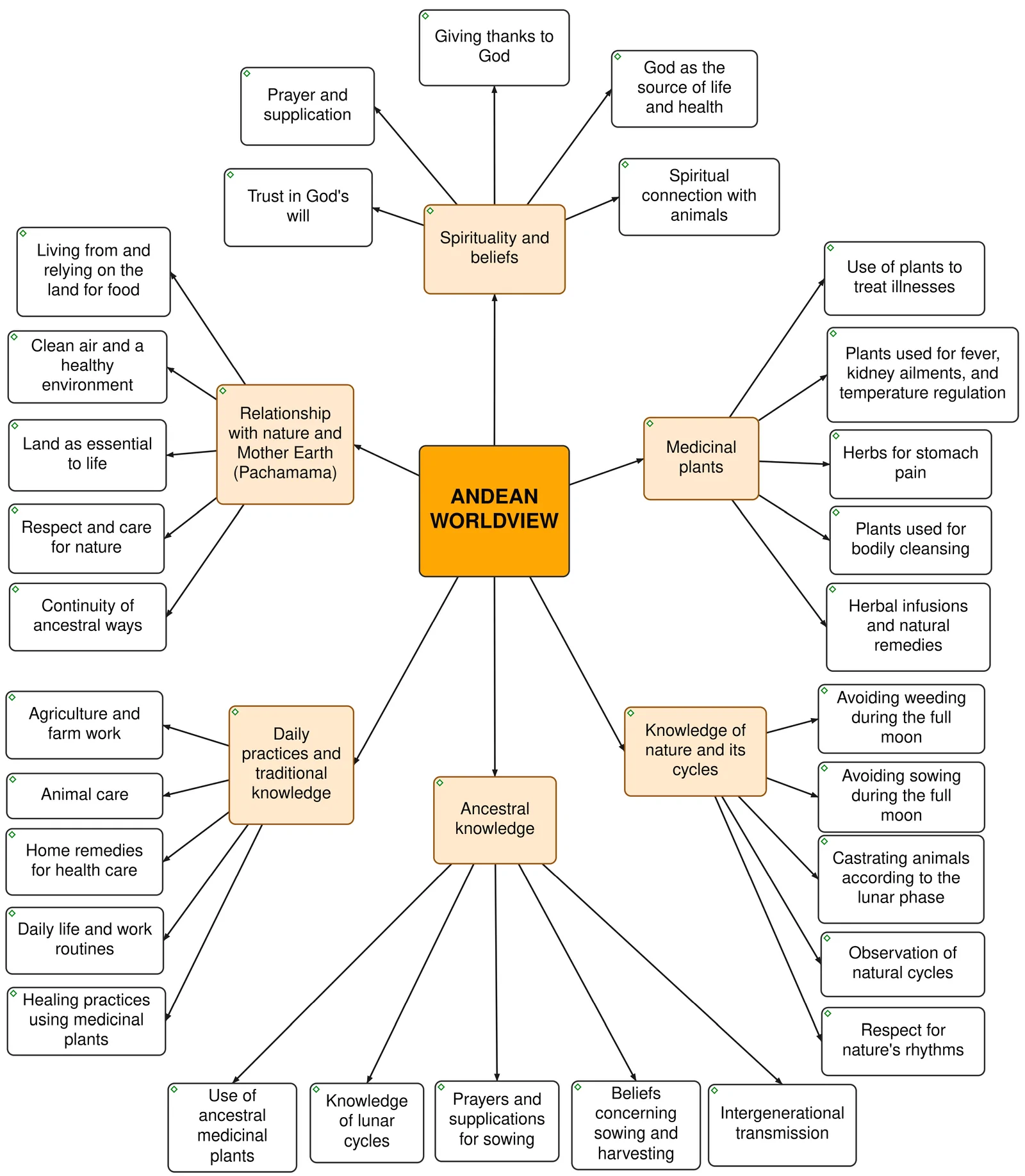
Category 3. Semantic network of Andean worldview.

The process was supported by ATLAS.ti software (version 23, ATLAS.ti Scientific Software Development GmbH, Berlin, Germany) [35], used to store transcriptions, organize codes, retrieve fragments, and maintain analytical traceability; the construction and interpretation of themes were carried out by the research team, not by the software. Coding was performed by two individuals: the principal investigator and a nursing professional with a master’s degree in public health, who examined the transcriptions and cross-checked the codes with the original fragments. Interpretative differences were analyzed through reflective discussion and joint review of the narrative evidence until analytical agreements were reached. The semantic networks for the three analytical categories are presented in Appendix A: responsibility in health care (Figure A1), life habits (Figure A2), and Andean worldview (Figure A3).

### 2.6. Methodological Rigor and Reflexivity

Methodological rigor was addressed using the criteria of credibility, transferability, dependability, and confirmability proposed by Lincoln and Guba [29]. The study was reported following the COREQ [36] and SRQR [37] guidelines, which are relevant to interview-based qualitative research and the comprehensive reporting of study design, context, reflexivity, participant selection, analysis, findings, and limitations. 

Credibility was addressed as a cultural contextualization strategy; preliminary findings were compared with five key informants from Pijal who were not part of the interviewed group. They were presented with an anonymized synthesis of the categories to assess their clarity, relevance, and cultural resonance. This procedure did not constitute a return of results to the 20 participants, but rather a complementary cultural contrast strategy. The key informants did not code the transcripts or make final analytical decisions. Community members did not participate in manuscript preparation.

Subsequently, triangulation was carried out with three external analysts—two researchers with experience in qualitative methodology in intercultural health and an anthropologist with a background in Andean studies—who were independent of the research team. They reviewed the transcripts, codes, and emerging themes, and their interpretations were contrasted with those of the research team until consensus was reached. The cultural contrast with key informants and the external analytical review did not alter the three overarching categories; however, these procedures contributed to refining the wording of several subthemes, clarifying the relationships among categories, and strengthening the cultural contextualization of the interpretations. Final analytical decisions remained the responsibility of the research team.

Transferability was based on an in-depth description [26,29] of the community context, which included the geographic, cultural, social, and economic characterization of the community of Pijal, as well as a detailed description of the participants and the research process; this description allows other researchers to assess the applicability of the findings in similar contexts.

Dependability was strengthened through systematic documentation of methodological and analytical decisions, including the external audit that enabled researcher triangulation and the traceability that the use of ATLAS.ti allowed over the coding process, enabling an assessment of the coherence and traceability of the entire process [29].

Confirmability was supported by the systematic preservation of recordings, anonymized transcriptions, codes, successive versions of subthemes and themes, and the narrative fragments that supported each interpretation. This documentation allowed an explicit and traceable relationship to be maintained between the participants’ voices and the findings presented [30].

Reflexivity was addressed through recognition of the interviewer’s position, considering that her nursing training, prior knowledge of health promotion, and affiliation with a university research team could influence question formulation, interaction with participants, and interpretation of the narratives. To foster a culturally situated understanding, participants’ language was prioritized, and their experiences were examined within their family, community, cultural, and territorial context [38].

Participants may have perceived the interviewer as an external health professional because of her nursing training and university affiliation. This perception may have influenced their willingness to share certain experiences, especially those related to non-biomedical health practices. To mitigate this effect, the exploratory nature of the study was emphasized, and participants were informed that their cultural practices would not be judged. The relationship between the researcher and the participants, although managed through informed consent and anonymity guarantees, implied an epistemic asymmetry that may have influenced responses. To address this limitation, during the interviews a non-directive conversational style was established, and questions were formulated based on explicit recognition of the participants’ local knowledge. During analysis, codes and themes were compared with key informants to verify that interpretations reflected their meanings, rather than categories imposed by the researcher’s biomedical training.

### 2.7. Ethical Considerations

The protocol was approved by the Human Research Ethics Committee of the Universidad Técnica de Manabí (CEISH-UTM-EXT_25-11-18_KVJJ, 19 January 2026) prior to recruitment and data production, which was conducted between March and June 2026. Administrative authorization and community consent were also obtained before contacting potential participants. The authors’ other affiliated institutions did not require additional ethical approval, acknowledgement, or exemption, because they did not participate as recruitment or data production sites. The community authorities requested that their names and identifying data not be disclosed, in order to protect their confidentiality; for this reason, the manuscript identifies only the instances that granted authorization: the parish GAD and the community council. The study adhered to the Declaration of Helsinki [39] and the CIOMS guidelines [40].

Before each interview, Katty-Elizabeth Cabascango verbally explained the study and provided the informed consent document. Each participant had the opportunity to ask questions and signed the consent form before the recording began. The document described the purpose, procedures, risks, confidentiality, the use of anonymized quotations, and the right to omit questions, interrupt or end the interview, and withdraw without consequences. Participation was voluntary.

The information was stored in a digital database on the principal investigator’s computer, with restricted access exclusively to the research team, in compliance with the principles of confidentiality, security, and limited retention established by the Ecuadorian Organic Law on Personal Data Protection [41]. The identity and personal data of participants were further protected through alphanumeric codes (P1 to P20), preserving anonymity in the presentation of results; the audio recordings and transcriptions will be kept only for the time necessary for the purposes of this study, after which they will be destroyed. Likewise, measures were implemented to avoid any physical, psychological, or emotional impact derived from participation.

Narratives about medicinal plants, spirituality, rituals, and ancestral knowledge were treated as culturally situated knowledge, used exclusively for the purposes authorized by the participants within the framework of this study. Data handling also complied with the ethics codes established by the International Council of Nurses (ICN) [42].

## 3. Results

### 3.1. Sociodemographic Characteristics of Participants

The sample comprised 20 Kichwa Kayambi adults from the Pijal community: 15 women (75%) and 5 men (25%). Eight participants were aged 25–39 years, eight were aged 40–59 years, and four were aged 60 years or older. All resided in the rural community. To reduce the risk of deductive disclosure, their sociodemographic characteristics are reported only in aggregate and cannot be linked to the quotation codes (Table 1).

### 3.2. Lived Experiences and Perceptions Related to Healthy Lifestyles

Lived experiences bring together the concrete actions, routines, and experiences through which participants care for their health in daily life. Thematic analysis of the interviews identified three interrelated categories: responsibility in health care, life habits, and Andean worldview. These categories integrate participants’ daily experiences and the cultural meanings attributed to a healthy life. The findings show that health care is not conceived exclusively as an individual behavior, but as a practice linked to economic conditions, family and community relationships, work, spirituality, and territory. The emerging categories and subcategories, along with the corresponding illustrative quotes, are presented in Table 2.

The semantic networks developed for the three analytical categories—responsibility in health care, life habits, and Andean worldview—are presented in Appendix A (Figure A1, Figure A2 and Figure A3).

#### 3.2.1. Responsibility in Health Care

The category of responsibility in health care describes the way in which participants recognize their active involvement in maintaining well-being. This responsibility is expressed through daily decisions related to diet, disease prevention, and timely attention to health alterations. However, possibilities for self-care are conditioned by available economic resources and family and community support.

Participants related self-care to the organization of meal schedules and the selection of foods considered nutritious. One participant explained: “I have breakfast at 6, lunch at 2, and dinner at 7 or 8 at night” (P11). Another participant pointed out that a healthy diet consists of “eating a balanced diet of proteins and vegetables” (P14). These narratives show that responsibility for health is materialized in practices embedded in household dynamics and not only in knowledge of health recommendations.

The accounts also evidenced that knowing about a healthy practice does not guarantee the possibility of maintaining it. Economic constraints appeared as a barrier to acquiring certain foods: “We don’t have enough money to buy vegetables and follow the diet” (P13). Unemployment and income instability limited the capacity to sustain some nutritional recommendations, revealing a tension between the will for self-care and material living conditions.

In the face of these limitations, community organization was perceived as a protective resource. The expression “It’s very quiet here… we are united” (P17) reflects the importance attributed to safety, coexistence, and mutual support. Therefore, responsibility for care is not configured as an exclusively individual obligation, but as a shared process among the individual, the family, and the community.

#### 3.2.2. Life Habits

The life habits category encompasses the daily practices through which participants seek to maintain their physical, emotional, and social well-being. These practices include physical activity, diet, rest, family coexistence, social relationships, and culturally situated ways of using tobacco and alcohol.

Physical activity was mainly integrated into agricultural, domestic, and community labor. Participants did not identify it solely with scheduled exercise, but also with walking, working the land, jogging, or playing soccer. The statement “What we do most is walk, work, and go for a jog” (P14) shows the continuity between bodily movement and productive life. The intensity and frequency of these activities varied according to age, occupation, and family responsibilities.

Healthy eating was interpreted from two sources of knowledge. On the one hand, participants incorporated recommendations provided by health professionals, such as reducing salt and sugar consumption and increasing vegetable intake. On the other hand, they attributed special value to foods produced within the community: “We have watercress, turnip here… we grow Swiss chard… nothing is treated [with chemicals]… for me it’s healthy food” (P17). The expression “not treated” is used to differentiate local products perceived as natural from those associated with chemicals or industrial processes. Thus, healthy eating is related to the origin of food, family production, and the bond with the territory.

Rest was conditioned by work schedules and domestic responsibilities. The narratives described days that begin early and, in some cases, reduce the time available for sleep. Sleep did not appear as an isolated practice but as part of a daily organization determined by agricultural work, animal care, and family activities.

Emotional well-being was linked to spirituality, family coexistence, and contact with the natural environment. One participant stated: “When I feel tired, I read the Bible, I feel good” (P05). Another noted: “Being together with my children makes me feel good; in our free time we go out to the countryside” (P09). These experiences show that emotional recovery occurs through simple daily practices, such as prayer, family accompaniment, and spending time in natural spaces.

Dialogue and family support were also recognized as resources for preventing conflicts and coping with difficulties. The statement “Dialogue in the family is the best way to avoid problems” (P13) shows that well-being is built through relationships of trust, communication, and accompaniment. The family thus functions as a space for care, knowledge transmission, and emotional regulation.

Accounts of tobacco and alcohol revealed ambivalent meanings. Participants recognized the harmful effects of habitual consumption and the possibility of intergenerational reproduction of these behaviors. Nevertheless, they also described specific ritual or medicinal uses: “They do the cleansing with a cigarette or with alcohol” (P14) and “The puntas [are used] for the flu” (P20). This tension shows that the meaning of the same substance depends on its purpose, frequency, and the cultural context of use: it may be perceived as harmful in daily consumption and as a legitimate resource within a ritual or medicinal practice.

Finally, social relationships enabled the articulation of biomedical knowledge with traditional knowledge. Guidance from health personnel was valued for facilitating prevention and timely care, while the experience of elders retained legitimacy within the community. One participant synthesized this complementarity: “Without modern medicine… we wouldn’t know exactly what we had, but the old ways also help you a lot” (P14). Life habits are therefore not constructed from a single knowledge system. They are shaped through interaction among professional recommendations, family experience, knowledge transmitted by elders, and the concrete conditions of community life.

#### 3.2.3. Andean Worldview

The Andean worldview category encompasses the meanings that relate health to spirituality, nature, agricultural work, medicinal plants, and knowledge transmitted across generations. From this perspective, a healthy life implies preserving balance among the individual, family, community, territory, and the spiritual world.

Spirituality formed part of daily care practices. Prayer and gratitude accompanied the start of activities: “In the morning we ask God that everyone is well” (P04). Another participant expressed: “Being with the animals; spiritually, God gives us life and health” (P11). Although the narratives mainly included Christian references, spirituality was also related to animals, the land, work, and the continuity of life. These accounts show that the spiritual dimension is not experienced separately from daily activities. Faith is integrated into home care, agricultural work, and the relationship with the environment, functioning as a source of emotional strength, hope, and meaning.

Pachamama was recognized as a source of life, food, work, and well-being. The expression “We follow the path of the grandparents who lived by feeding themselves from the field” (P04) links current practices with the memory of previous generations. Likewise, the statement “The land is so valuable for everything” (P06) reflects its importance, both material and symbolic. Rural life was perceived as quieter and healthier than urban life, due to contact with nature, the availability of local foods, and air quality. However, this valuation coexisted with economic and labor transformations, such as employment in floriculture and unemployment, which may limit the continuity of family agricultural practices.

The use of medicinal plants constituted a habitual domestic care practice. Participants mentioned lemon, plantain, caballo chupa, linseed, linden, chamomile, nettle, guava, paiteña onion, black nightshade, and anguyuyo to treat fever, digestive complaints, kidney or urinary problems, and other ailments. This knowledge is grounded in family and community experience and is transmitted through observation and practice. In this study, medicinal uses are presented as experiences and meanings expressed by the participants, without attributing clinical efficacy to them or proposing them as substitutes for assessment by health professionals.

Ancestral knowledge also encompassed knowledge related to sowing, harvesting, lunar cycles, childbirth, and animal care. The statement “In a full moon… you should not plant” (P09) shows how observation of natural cycles guides certain productive decisions. Likewise, spiritually requesting a good harvest expresses the relationship between faith, work, and nature. Agriculture, food and remedy preparation, and animal care constitute daily spaces in which this knowledge is practiced, acquires meaning, and is transmitted across generations. Therefore, ancestral knowledge is not preserved merely as memories of the past, but as knowledge that continues to guide some decisions in community life.

Although most participants agreed on the categories presented, some individual variations were identified. For example, two participants (P02 and P15) reported difficulties in maintaining a healthy diet due to lack of time from working in floriculture, unlike those who were exclusively engaged in family agriculture. Likewise, one participant (P08) expressed not using medicinal plants, preferring exclusively conventional medical care. These variations enrich the understanding of the community’s internal diversity.

In response to the study objective, the findings allow us to understand that the lived experiences and perceptions of healthy lifestyles are configured within a fabric of personal, family, community, cultural, and territorial relationships. Individual responsibility acquires meaning within economic possibilities, which can facilitate or limit access to food and care; habits are sustained through work, family, and community; and the Andean worldview links health with spirituality, the land, and the memory of elders.

The three categories are interrelated and manifest simultaneously in actions such as cultivating food, walking during agricultural labor, preparing medicinal plants, engaging in family dialogue, and giving thanks for the harvest. This articulation constitutes the interpretative core of the study: a healthy life is understood as an integral experience in which personal care, community coexistence, cultural knowledge, and the relationship with the territory converge.

## 4. Discussion

The central contribution of this study is to demonstrate that, for Kichwa Kayambi adults from Pijal, a healthy life does not constitute a sum of isolated behaviors, but rather a relational practice situated within material conditions and bonds with family, community, spirituality, and territory. This interpretation extends studies that describe multiple dimensions of Indigenous health [4,5] by explaining how these dimensions converge in everyday decisions and how they may guide culturally congruent nursing actions.

Personal responsibility appeared conditioned by access to food, employment, and the organization of care. Therefore, the distance between knowing a recommendation and being able to apply it should not be interpreted as a lack of motivation. This finding converges with research on food risk, insecurity, and inequality in Indigenous and rural populations [10,13,18], but adds that community cohesion functions as a care resource in the face of structural constraints. Similarly, walking, working the land, or caring for animals exceeds the biomedical separation between exercise, work, and domestic life; movement acquires meaning within subsistence, reciprocity, and territory [4,9].

The narratives also did not absolutely oppose biomedical and ancestral knowledge. Recommendations regarding salt [4], sugar, vegetables, and timely care coexisted with the valuation of local foods, medicinal plants, and the knowledge of elders [14,16]. This coexistence does not imply equivalence of clinical efficacy. Rather, it reveals a space of negotiation in which nursing must recognize the cultural meaning of a practice and, simultaneously, communicate clearly its benefits, uncertainties, and risks. The same logic applies to the ritual or domestic uses of tobacco and alcohol: understanding their context does not require omitting harm prevention [7].

Leininger’s Theory of Culture Care Diversity and Universality makes it possible to translate these findings into three modes of action [19,20]. First, cultural preservation or maintenance is pertinent for resources that sustain well-being without known harm, such as family support, the minga, everyday spirituality, movement incorporated into community activities, and the bond with the land. Preserving does not mean idealizing or assuming homogeneity, but rather recognizing resources valued by the participants and supporting their continuity when desired [13,14,15].

Second, cultural accommodation or negotiation can guide dialogue on nutrition, rest, access to services, and the use of medicinal plants. In these domains, professionals and the community can agree upon practices compatible with economic possibilities, work rhythms, and cultural preferences, verify interactions or risks, and keep biomedical care pathways open [20]. The assertion that modern medicine makes it possible to know “exactly” what is happening, together with the value attributed to the “old ways,” exemplifies this practical articulation and also aligns with Pender by highlighting prior experiences and situational and interpersonal influences [21,22].

Third, cultural repatterning or restructuring is pertinent when a practice may produce harm, for example, if a belief about a remedy delays diagnosis or replaces oncological treatment, or if the use of tobacco and alcohol is normalized outside a ritual context. Repatterning must be constructed without disauthorizing the individual or stigmatizing community knowledge: it requires explaining the evidence, recognizing the meaning attributed to the practice, and agreeing upon safe alternatives. Nevertheless, economic and occupational barriers are not cultural behaviors that should be “corrected”; they require structural responses, effective access to food and services, and policies that strengthen community capacities.

This reading also avoids treating the Andean worldview as a fixed repertoire. Differences among generations, occupations, and therapeutic preferences show an internally diverse community in transformation. Pachamama and Sumak Kawsay articulate territory, memory, and well-being, but their everyday expression coexists with floriculture, unemployment, biomedical care, and changes in family agriculture. Cultural congruence therefore requires asking and negotiating with each person and family, rather than attributing practices to them on the basis of their ethnic belonging.

Four limitations should be considered. The study was based on individual interviews without participant observation, and therefore may have omitted non-verbalized practices and did not examine group interactions. The initial target of 20 participants and the purposive sampling with chain referrals favored diversity and access, but may have overrepresented networks connected to the three entry points; no statistical generalization is intended. The findings come from a single community, and their transferability requires assessing contextual similarities. Finally, conducting the interviews in Spanish may have limited nuances expressible in Kichwa; the translation of quotations into English adds another interpretive layer, mitigated through verification of conceptual equivalence.

Because women comprised 75% of the sample, experiences related to household, family, and community care may have received greater analytical visibility, whereas men’s experiences may be underrepresented. The findings should therefore not be interpreted as representing equivalent perspectives across sex or gender roles.

Future studies could combine interviews with participant observation, deliberately incorporate more men, and contrast generations and communities. Participatory research with Kichwa speakers would also make it possible to examine how language shapes the meanings of care and to co-produce interventions based on Leininger’s modes of action.

## 5. Conclusions

This study provides a situated interpretation of healthy living among Kichwa Kayambi adults from Pijal: care is produced in the interdependence between personal capacity, material conditions, family and community relationships, spirituality, and territory. Its distinctive contribution lies in demonstrating how these dimensions converge in everyday practices and cannot be reduced to individual choices or biomedical indicators.

For transcultural nursing, the findings offer concrete guidance: to preserve safe and valued community resources; to accommodate or negotiate practices at the intersection of knowledge systems; and to repattern those that may cause harm, through respectful dialogue and clinical evidence. This application must be accompanied by responses to economic and access barriers, without homogenizing the community or converting structural problems into individual responsibilities.

## Figures and Tables

**Table 1:** Aggregated sociodemographic characteristics of participants (*n* = 20).

Characteristic	Category	*n* (%)
Age group	25–39 years	8 (40%)
	40–59 years	8 (40%)
	≥60 years	4 (20%)
Sex	Female	15 (75%)
	Male	5 (25%)
Educational level	No formal education or incomplete basic general education	8 (40%)
	Completed basic general education	3 (15%)
	Completed high school	6 (30%)
	Higher education	3 (15%)
Main occupation	Household work	9 (45%)
	Agriculture	7 (35%)
	Craft work or currently unemployed	4 (20%)

Note: Characteristics are reported in aggregate to reduce the risk of deductive disclosure. The data in this table cannot be linked to the quotation codes in Table 2.

**Table 2:** Categories, subcategories, and illustrative quotes emerging from the lived experiences and perceptions of Indigenous adults in the community of Pijal.

Categories	Subcategories	Illustrative Quotes
Responsibility in health care	Responsibility and self-care	“I have breakfast at 6, lunch at 2, and dinner at 7 or 8 at night.” (P11); “Eating a balanced diet of proteins and vegetables.” (P14); “Don’t mix food, for example, pasta with potatoes, don’t mix because that makes you gain weight, that only makes you bloated.” (P15).
	Barriers and facilitators	“We don’t have enough money to buy vegetables and follow the diet.” (P13); “Here with unemployment, there isn’t much work. They still dedicate themselves to agriculture, more than floriculture.” (P16); “It’s very quiet here, we walk at night, because the commune is large and we are united and there is no danger, because if something comes in here, we punish it together with the commune.” (P17).
Life habits	Physical-sports activity	“Exercising and walking every day, three hours daily.” (P4); “What we do most is walk, work, and go for a jog.” (P14); “Now only with work, playing soccer and walking a little.” (P16).
	Social relationships	“The doctors, through talks, saying that they should get checked, treat any illness in time.” (P12); “People from before didn’t get sick like now, they ate everything ripened from the field.” (P13); “I think they would go together, because without modern medicine, I think we wouldn’t know exactly what we had, but the old ways also help you a lot.” (P14).
	Tobacco and alcohol consumption	“I have seen them do the limpia [ritual cleansing], as they call it, they do the cleansing with a cigarette or with alcohol.” (P14); “I think that if your father smokes, your son when he grows up or as an adult will also smoke.” (P15); “They use alcohol, the pure liquor we call it, las puntas, for the flu.” (P20).
	Meanings of healthy eating	“I eat vegetables because the doctors told me to eat only vegetables, to eat without salt, without sugar.” (P1); “My health has changed through my nutritious diet, the truth is I gained quite a bit of weight and now I’m returning to normal, eating, consuming vegetables.” (P3); “We have watercress, turnip here. We also grow Swiss chard, nothing is treated [with chemicals], we have beans, all corn, so for me it’s healthy food.” (P17).
	Daily rest and sleep	“In the morning I get up at 6 in the morning and in the afternoon I go to sleep at 8.” (P3); “I go to sleep at 11 or 10, I wake up at 4 in the morning.” (P11); “I go to bed at ten at night. And I wake up at five-thirty in the morning.” (P16).
	Emotional well-being	“When I feel tired, I read the Bible, I feel good.” (P5); “Being together with my children makes me feel good. In our free time we go out to the countryside.” (P9); “It means a lot to me to have a healthy life. I think it freed me from stress because I felt bad, as I say, with myself.” (P14).
	Family support	“Providing information about everything regarding diet, regarding tobacco consumption, to avoid diseases.” (P9); “Sometimes families get together, give talks, prepare home remedies.” (P12); “Especially in the way of living, dialogue in the family is the best way to avoid problems.” (P13).
Andean worldview	Spirituality and beliefs	“In the first place, we always ask God in the morning that we are all well and everything like that. In fact, from the work I do, I feel good.” (P4); “First of all, give thanks to God, you have to pray, you have to make requests and then it is his will.” (P8); “Being with the animals, spiritually, God gives us life and health.” (P11).
	Medicinal plants	“Here with the medicinal plants from the field we heal when the kidneys hurt, when there is a fever. We use lemons, plantain, caballo chupa, linseed.” (P1); “I use linden, chamomile, nettle, guava to drink and roasted paiteña onion for stomach pain.” (P6); “Black nightshade is good for cleansing the body, even for urinary infections, when doing evaporation or washing. Chamomile is also for consumption. Anguyuyo is for fever.” (P13).
	Relationship with nature and Mother Earth (Pachamama)	“Our grandparents have lived their whole lives working on the land and we follow the path of the grandparents who lived by feeding themselves from the field.” (P4); “Actually, here in the countryside it is quieter than living in the city, because the air is healthier.” (P5); “The land is so valuable for everything, because if the land didn’t exist, nothing would exist.” (P6).
	Daily practices and traditional knowledge	“I dedicate myself to agriculture. The others work in flowers [floriculture].” (P17); “I cured my [health condition] with watercress, drinking juice for 12 mornings on an empty stomach. […] It is crushed and the pure juice is taken.” (P18). [Authors’ note: The specific diagnoses have been omitted to protect participant confidentiality. This account reflects the participant’s belief and does not establish that watercress is an effective treatment. Health conditions require appropriate clinical assessment and care.] “In the morning, the normal thing, the typical thing, I start making breakfast for my children, then I go with the animals, I come back, I also dedicate myself to the animals that are in the house.” (P20).
	Ancestral knowledge	“We say here with pacunga and chamomile, for example, chamomile is for stomach pain, for young ladies who are pregnant, to hasten labor pain, boiled water with pacunga and coriander seed is good.” (P4); “It is not good to weed during a full moon, also you should not plant during a full moon. In a new moon it is bad to castrate animals.” (P9); “Asking God when we start sowing to give us a good harvest.” (P12).

## Data Availability

The data generated and analyzed during the present study are available from the corresponding author upon reasonable request, provided that access is compatible with ethical considerations, confidentiality protection, and safeguarding of individual and community identity. Due to the territorial and intercultural nature of the study, the complete database is not publicly available.

## Supplementary Materials

The following supporting information can be downloaded at: https://www.mdpi.com/article/10.3390/healthcare14193250/s1, Supplementary Material S1: The 32-item COREQ checklist; Supplementary Material S2: The semi-structured interview guide used in the study.
