# Supplementary material for: Lived Experiences and Perceptions of Healthy Lifestyles Among Kichwa Kayambi Indigenous Adults: An Interpretative Qualitative Study in the Community of Pijal
Source: Healthcare (Basel). 2026 Oct 1;14(19):3250. doi: 10.3390/healthcare14193250 (PMC13665196; doi:10.3390/healthcare14193250)
Supplement: Supplementary file 1 [file healthcare-14-03250-s001.zip › healthcare-4543665 Supplementary_Material_S1_The 32-item COREQ checklist.pdf]

Supplementary Material S1: The 32-item COREQ checklist

Manuscript: Lived Experiences and Perceptions of Healthy Lifestyles Among Kichwa Kayambi Indigenous Adults: An Interpretative Qualitative Study in the Community of Pijal

Manuscript ID: healthcare-4543665

This completed checklist identifies where each COREQ item is addressed in the second proof of manuscript healthcare-4543665. Page numbers refer to that manuscript. Where an item was not undertaken or not fully reported, this is stated explicitly.

.

Domain and topic	Item	COREQ guide question or description	Information reported in the manuscript	Page
**Domain 1: Research team and reflexivity Personal characteristics**	**1**	Interviewer/facilitator — Which author/s conducted the interview or focus group?	All interviews were conducted by K.-E.C.	5
	**2**	Credentials — What were the researcher's credentials? e.g. PhD, MD	K.-E.C. is identified as a woman nursing professional and member of the research team, trained in qualitative methodology and semi-structured interviewing. K.J.-J. is identified as the woman principal investigator.	5
	**3**	Occupation — What was their occupation at the time of the study?	K.-E.C. is reported as a nursing professional; K.J.-J. is reported as the principal investigator. The coding team included the principal investigator and a nursing professional with a master's degree in public health.	5-6
	**4**	Gender — Was the researcher male or female?	The manuscript reports that K.-E.C. and K.J.-J. are women.	5
	**5**	Experience and training — What experience or training did the researcher have?	K.-E.C. received training in qualitative methodology and semi-structured interviewing through in-person workshops. The manuscript also describes the qualitative and intercultural expertise of the external analysts.	5-7
**Relationship with participants**	**6**	Relationship established — Was a relationship established prior to study commencement?	Prior to the study, K.-E.C. had no familial, occupational, educational, or caregiving relationship with participants.	5
	**7**	Participant knowledge of the interviewer — What did the participants know about the researcher? e.g. personal goals, reasons for doing the research	Initial contacts with participants and authorities were used to explain the study before the interviews. The interviewer had no prior caregiving or other relationship with participants.	5,7
	**8**	Interviewer characteristics — What characteristics were reported about the interviewer/facilitator? e.g. bias, assumptions, reasons and interests in the research topic	The manuscript discusses the interviewer's gender, nursing training, university affiliation, possible positioning as an external health professional, potential epistemic asymmetry, and the non-directive and culturally respectful strategies used to mitigate these influences.	5, 7
**Domain 2: Study design Theoretical framework**	**9**	Methodological orientation and theory — What methodological orientation was stated to underpin the study? e.g. grounded theory, discourse analysis, ethnography, phenomenology, content analysis	Interpretative qualitative design guided by interpretive ethnography and naturalistic inquiry, with hybrid thematic analysis. Leininger's Culture Care Diversity and Universality Theory and Pender's Health Promotion Model were used as sensitizing concepts rather than fixed analytical categories.	3,6
**Participant selection**	**10**	Sampling — How were participants selected? e.g. purposive, convenience, consecutive, snowball	Snowball or chain-referral sampling was used. Recruitment began from three deliberately diverse entry points to reduce dependence on a single social network.	4
	**11**	Method of approach — How were participants approached? e.g. face-to-face, telephone, mail, email	Potential participants were approached through three community entry points (a community leader, an older adult woman, and a young farmer), followed by referrals. Administrative and community authorization preceded contact.	4
	**12**	Sample size — How many participants were in the study?	Twenty Kichwa Kayambi adults participated.	4
	**13**	Non-participation — How many people refused to participate or dropped out? Reasons?	Twenty eligible individuals were invited; all accepted and completed the interview. There were no refusals, withdrawals, or dropouts.	4
**Setting**	**14**	Setting of data collection — Where was the data collected? e.g. home, clinic, workplace	Interviews took place in homes, community spaces, or workplaces, according to participant agreement and with attention to privacy.	5

	**15**	Presence of non-participants — Was anyone else present besides the participants and researchers?	No. Each interview was conducted solely by K.-E.C. with the participant.	5
	**16**	Description of sample — What are the important characteristics of the sample? e.g. demographic data, date	The manuscript reports the sample's sociodemographic characteristics and data-production period. Sociodemographic characteristics are presented in aggregate in Table 1 and are not linked to the participant codes used for quotations.	4,8
**Data collection**	**17**	Interview guide — Were questions, prompts, guides provided by the authors? Was it pilot tested?	The manuscript reports the opening questions, use of probes, cultural adaptation, and specialist review of the guide. The complete semi-structured interview guide is provided as Supplementary Material S2. A formal pilot test is not reported.	4-5; Supplementary Material S2
	**18**	Repeat interviews — Were repeat interviews carried out? If yes, how many?	No repeat interviews were conducted.	5
	**19**	Audio/visual recording — Did the research use audio or visual recording to collect the data?	Interviews were audio-recorded with prior authorization.	5
	**20**	Field notes — Were field notes made during and/or after the interview or focus group?	After each interview, K.-E.C. recorded contextual, nonverbal, and reflective field notes.	5
	**21**	Duration — What was the duration of the interviews or focus group?	Each interview lasted 60–90 minutes.	5
	**22**	Data saturation — Was data saturation discussed?	The target of 20 interviews was predetermined as a sampling-design decision. Concurrent analysis assessed corpus adequacy; final conversations reiterated previously identified patterns without substantial variation. The manuscript does not retrospectively present the sample size as determined by saturation or information power.	4
	**23**	Transcripts returned — Were transcripts returned to participants for comment and/or correction?	No. Transcripts were not returned to participants. Fidelity was checked by two researchers who re-listened to the original recordings and verified each transcript.	5-6

Domain and topic	Item	COREQ guide question or description	Information reported in the manuscript	Page
**Domain 3: Analysis and findings**<br>**Data analysis**	**24**	Number of data coders — How many data coders coded the data?	Two individuals coded the data: the principal investigator and a nursing professional with a master's degree in public health. Codes were cross-checked against original excerpts and interpretive differences were resolved through reflective discussion.	6
	**25**	Description of the coding tree — Did authors provide a description of the coding tree?	The manuscript describes the progression from codes to subthemes and three overarching categories. Semantic networks for the three categories are presented in Appendix A, Figures A1–A3.	6, 18-20
	**26**	Derivation of themes — Were themes identified in advance or derived from the data?	Themes and interpretive relationships were developed through a hybrid thematic approach: sensitizing concepts informed the initial orientation, while codes, subthemes, themes, and relationships were constructed from participants' narratives.	6
	**27**	Software — What software, if applicable, was used to manage the data?	ATLAS.ti version 23 was used to store transcripts, organize codes, retrieve excerpts, and maintain analytical traceability. Theme construction and interpretation were performed by the research team.	6
	**28**	Participant checking — Did participants provide feedback on the findings?	No formal participant checking was undertaken with the 20 interviewees. Five Pijal key informants who were not interview participants reviewed an anonymized synthesis for clarity, relevance, and cultural resonance. This cultural contrast and external analytical review refined subtheme wording and relationships without altering the three overarching categories; final analytical decisions remained with the research team.	6-7
**Reporting**	**29**	Quotations presented — Were participant quotations presented to illustrate the themes/findings? Was each quotation identified? e.g. participant number	Yes. Illustrative quotations are presented in Table 2 and throughout the Results. Quotations are identified with anonymized participant codes (P1–P20).	8–15
	**30**	Data and findings consistent — Was there consistency between the data presented and the findings?	Yes. Each category and subcategory is supported by participant quotations, and the analytical account is explicitly linked to the coded narrative material.	8–15
	**31**	Clarity of major themes — Were major themes clearly presented in the findings?	Yes. Three interrelated categories are clearly identified: responsibility in health care, life habits, and Andean worldview.	11-15
	**32**	Clarity of minor themes — Is there a description of diverse cases or discussion of minor themes?	Subcategories and divergent accounts are reported within the major categories. The Results explicitly describe variations related to floriculture work, use of medicinal plants, and preference for conventional care.	8–15

Checklist source : Tong et al. [36]. The full bibliographic reference is provided in the main manuscript.
