# Supplementary material for: Lived Experiences and Perceptions of Healthy Lifestyles Among Kichwa Kayambi Indigenous Adults: An Interpretative Qualitative Study in the Community of Pijal
Source: Healthcare (Basel). 2026 Oct 1;14(19):3250. doi: 10.3390/healthcare14193250 (PMC13665196; doi:10.3390/healthcare14193250)
Supplement: Supplementary file 1 [file healthcare-14-03250-s001.zip › healthcare-4543665 Supplementary_Material_S2_Interview_Guide.pdf]

Supplementary Material S2: The semi-structured interview guide used in the study

Study Topic: Lived Experiences and Perceptions of Healthy Lifestyles among Indigenous Adults from the Pijal Community, Imbabura, Ecuador, 2026.

General Objective: To explore and gain an in-depth understanding of the lived experiences and perceptions of healthy lifestyles among Indigenous adults from the Pijal Community, Imbabura, Ecuador, in 2026.

Instructions: The guide contains 58 potential questions and probes organized into eight dimensions. It was used flexibly rather than administered as a fixed questionnaire. The interviewer selected questions and follow-up probes according to each participant's narrative; not all questions were asked in every interview, and their sequence could vary.

Ethical Considerations

Participation in this interview is voluntary and confidential. The interview is conducted in a respectful and culturally sensitive environment that encourages participants to express their experiences and perspectives freely, while acknowledging and valuing their cultural knowledge and traditional understandings of health and well-being.

Estimated Duration: Approximately 60–90 minutes, depending on the depth and course of each participant's narrative.

Sociodemographic Characteristics

1. How old are you (in completed years)?

………………………………………….

2. What is your sex?

1. Male
2. Female

3. What is your current place of residence?

1. Urban area
2. Rural area

4. What is your highest level of educational attainment?

1. No formal education / illiteracy
2. Completed basic general education
3. Incomplete basic general education

4. Completed upper secondary education
5. Incomplete upper secondary education
6. Higher education (technical/technological degree or bachelor's degree)

5. What is your cultural identity?

1. Kichwa Karanki
2. Kichwa Otavalo
3. Kichwa Kayambi
4. Kichwa Natabuela

6. What language(s) do you speak?

1. Kichwa
2. Spanish
3. Bilingual (Kichwa–Spanish)
4. Other: …………………………………….

7. What is your primary role or occupation within the community?

1. Agricultural worker
2. Artisan
3. Homemaker
4. Unemployed

DIMENSION 1: Individual Responsibility for Health Care

1. Could you tell me a little about yourself and the role you have within your community?
2. What do you do for a living, and what are the main occupations or activities of people in your community?
3. How would you describe the way of life in this community?
4. What activities do you usually engage in as part of your daily routine?
5. What activities or moments help you feel physically, emotionally, or spiritually well in your daily life and during your leisure time?
6. What health- or well-being-related concerns are currently present in your community?
7. Do you regularly engage in any form of physical activity, such as walking, working in the fields, dancing, or other activities? If not, could you explain the reasons why?
8. Could you tell me about any healthy habit or routine that you have recently incorporated into your life and how you were able to adopt it?

DIMENSION 2: Physical Activity and Exercise Habits

9. How do you currently take care of your health, and how has the way you care for yourself changed over time?
10. Was there any significant event or turning point in your life that motivated you to improve your health?
11. What inspired or motivated you to adopt a healthier way of living?
12. Were there any unhealthy habits that you stopped or changed? Was this difficult for you? How did you manage to make those changes?
13. Did you receive support from anyone when trying to improve your health, or was it primarily your own decision? How did this influence your process of change?

DIMENSION 3: Health-Related Habits within Social Relationships

14. Who first taught you or spoke to you about how to take care of your health?
15. How have your family members or community elders influenced the way you currently care for your health? Are there any recommendations, teachings, or customs that you still follow?
16. Do you consider this traditional knowledge valuable in your life today? Why or why not?
17. Are there people close to you, such as family members, friends, or community leaders, who encourage or support you in maintaining healthy habits? How do they provide this support?
18. What role does your family play in aspects of your health such as diet, rest, or physical activity?
19. Do you participate in community activities related to health or well-being? If so, which ones?

DIMENSION 4: Cultural Practices and Traditional Health Knowledge

20. What does living in balance or harmony with nature mean to you, and how do you relate this balance to your health and well-being?
21. What cultural, traditional, or ancestral practices do you continue to use to care for your health?
22. Do you use traditional medicine, medicinal plants, rituals, ceremonies, or other ancestral practices to prevent or treat illness? Could you tell me about these practices?
23. Which foods or traditional preparations do you consider healthy within your culture?
24. Are there celebrations, significant dates, or particular times in your community that are associated with health or well-being? For example, are certain phases of the moon, days of the week, or other culturally significant times considered appropriate for healing practices or health-related activities?

SECTION 4.1: Tobacco and Alcohol Use

25. Within your community, what meaning or significance is associated with the consumption of alcoholic beverages, for example during celebrations, rituals, or family gatherings?

26. Do you consider alcohol consumption to have any social, spiritual, or symbolic significance within your culture? Could you explain why?
27. What are your views on tobacco use, including cigarettes, tobacco leaves, traditionally prepared tobacco, or other forms?
28. Are tobacco or alcoholic beverages used for any medicinal, ritual, ceremonial, or traditional purposes within your community? Could you describe these practices?
29. How do you perceive the effects of alcohol or tobacco use on people's health and on family or community relationships?
30. Have patterns or practices related to tobacco or alcohol use changed in your community in recent years? What do you think has contributed to these changes?

DIMENSION 5: Healthy Eating Habits

31. What aspects do you find most challenging when trying to maintain a healthy diet or a healthy lifestyle?
32. What do you usually do when you are unable to maintain healthy habits, such as eating well, getting adequate rest, or engaging in physical activity?
33. What strategies have you used to overcome these challenges?
34. Who or what has been most helpful in supporting you to maintain your health and well-being?
35. Have you received support from healthcare professionals, community health workers, or other institutions? If so, what type of support did you receive, such as consultations, home visits, educational sessions, or workshops?
36. What was the most useful or meaningful thing you learned from them?

DIMENSION 6: Psychological Health Habits

37. What does having a healthy lifestyle and feeling a sense of balance in your daily life mean to you?
38. How do you feel when you take care of your health?
39. Do you believe that living a healthy life also benefits your community? If so, in what ways?
40. Based on your own experiences, knowledge, or traditions, what advice would you give to young people in your community about how to take care of their health?

DIMENSION 7: Andean Worldview

41. Do you consider the traditional knowledge and wisdom of your ancestors to be valuable in relation to health and well-being? Why?
42. What health-related teachings or practices passed down by your grandparents or ancestors do you continue to apply in your daily life?
43. How would you describe your relationship with the land and the natural environment?

44. Do you spend part of your free time caring for or working the land? If so, could you describe these activities?
45. Do you feel that your own well-being is connected to the well-being of nature? In what way?
46. What practices do you engage in to maintain harmony with **Pachamama (Mother Earth)**?
47. Do you follow any customs, rituals, or traditional practices to express gratitude to or ask permission from the land before activities such as planting or harvesting? Could you describe them?
48. Do you use medicinal plants in your daily life?
49. Which medicinal plants do you commonly use to prevent or treat health problems, and how are they used?
50. Do you know of any ancestral or traditional remedies or preparations used to address specific ailments? Could you describe them?
51. Do you receive or use traditional treatments such as herbal baths, *limpias* (traditional cleansing practices), massages, or other treatments involving medicinal plants?
52. Do you believe that these traditional practices can complement conventional or modern healthcare? Why or why not?
53. For certain health concerns, do you place greater trust in Indigenous traditional medicine than in conventional or modern medicine? Could you explain the reasons for your preference?
54. Does your community perform rituals or ceremonies intended to promote healing, well-being, or protection among community members?
55. How do you personally participate in or contribute to these rituals or ceremonies aimed at promoting the health and well-being of the community?

DIMENSION 8: Daily Rest and Sleep Habits

56. Is there anything else you would like to share about your way of life or how you care for your health and well-being?
57. Could you describe your usual rest and sleep habits? For example, what time do you generally go to bed and wake up?
58. Is there any question that I have not asked but that you consider important for understanding your experiences, health practices, or way of life?
